# Measuring quality of life in opioid-induced constipation: mapping EQ-5D-3 L and PAC-QOL

**DOI:** 10.1186/s13561-016-0091-9

**Published:** 2016-04-21

**Authors:** Anthony James Hatswell, Stefan Vegter

**Affiliations:** 1 BresMed, 84 Queen Street, S1 2DW Sheffield, UK; 2 University College London, London, UK; 3 University of Groningen, Groningen, the Netherlands

## Abstract

**Background:**

In health economic evaluations, quality of life should be measured with preference-based utilities, such as the EuroQol 5 Dimension 3-level (EQ-5D-3 L). Non-preference-based instruments (often disease-specific questionnaires) are commonly mapped to utilities. We investigated if the relationship observed between the Patient Assessment of Constipation Quality of Life (PAC-QOL) and the EQ-5D-3 L in patients with chronic idiopathic constipation (CIC) also applies in opioid-induced constipation (OIC).

**Methods:**

EQ-5D-3 L patient-level data from a clinical study of lubiprostone in OIC (*n* = 439) were scored using the UK tariff. A published mapping between the PAC-QOL and the EQ-5D-3 L was tested using these data. New mapping formulas were analysed, including PAC-QOL total and subscale scores. The root mean square error (RMSE), the adjusted R^2^ and predicted/observed plots were used to test the fit.

**Results:**

The utility measured with the EQ-5D-3 L was 0.450 ± 0.329, with a distinctly bimodal distribution. This significantly improved if patients responded to treatment (defined as an increase of three spontaneous bowel movements per week, with no rescue medication taken). The published mapping in CIC performed poorly in this OIC population, and the PAC-QOL could not be reliably mapped on to the EQ-5D-3 L even when re-estimating coefficients. This was shown in our two mappings (using PAC-QOL total score, and subscale scores) by a high RMSE (0.317 and 0.314) and a low R^2^ (0.068 and 0.080), with high utilities underestimated and low utilities overestimated.

**Conclusions:**

Patients with OIC have a low quality of life which does improve with the resolution of symptoms. However the PAC-QOL cannot be used to estimate the EQ-5D-3 L utility – potentially as the PAC-QOL does not capture the all relevant aspects of the patients quality of life (for example the cause of the opioid use).

## Background

Although there are many potential causes of constipation, one of the most frequently reported is opioid usage: opioid-induced constipation (OIC). The condition is caused by opioids inhibiting the secretion of intestinal fluids and suppressing the peristaltic propulsion of the gastrointestinal tract, thereby slowing gastrointestinal motility [1]. This opioid effect causes a range of symptoms, from difficulty evacuating faeces to straining, hard stools, abdominal discomfort and bloating [2, 3].

Patient-reported quality of life in this disease area is low – a poster by Iyer et al. showed OIC patients with chronic non-cancer pain to have a baseline quality of life of approximately 0.45 using the EuroQol 5 Dimension 3-level (EQ-5D-3 L) [4]. Similar values were reported for a Dutch study, which estimated a median EQ-5D-3 L of 0.41 for constipated patients [5], although the cause of the constipation is not stated. Dunlop et al. reported that, using Short-Form 36 (SF-36) scores mapped to the EQ-5D-3 L in an OIC population with chronic non-cancer pain, patients had a utility of approximately 0.48 at baseline [6]. The existing literature suggests that the low utilities observed may arise from the comorbid conditions that necessitate long-term opioid therapy. This may also explain why a time trade-off exercise conducted with members of the UK general population (*n* = 308) showed a higher utility for OIC itself, rating the condition as having a utility of 0.74 [7].

In cost–utility analyses, when no preference-based instruments (such as the EQ-5D-3 L or Health Utilities Index) are available, mapping is a popular technique for predicting health state utilities. In mapping, the relationship between a non-preference-based instrument (often a disease-specific questionnaire containing aspects on quality of life) and a generic measure is estimated [8]. The Patient Assessment of Constipation Quality of Life (PAC-QOL) is a commonly used disease-specific questionnaire, which contains questions on worries and concerns, physical discomfort, psychosocial discomfort, and satisfaction [9]. Searching the Oxford Mapping Database, a study by Parker et al. reported a mapping between the PAC-QOL and the EQ-5D-3 L utility in chronic idiopathic constipation (CIC), but no report on mapping in OIC was found [10]. As CIC patients experience the same symptoms (with the same endpoints and scales used in clinical trials), our expectation was that a similar relationship would exist between the PAC-QOL and EQ-5D-3 L in OIC and CIC.

We investigated techniques for mapping PAC-QOL to the EQ-5D-3 L utilities for patients with OIC, including an exploration of the previously published mapping by Parker et al. [10].

## Methods

### Description of study 1033

The analyses presented in this article are based on data from Study 1033, which was a 12-week, double-blind, randomised study of lubiprostone (*n* = 219) compared to placebo (*n* = 220) [11]. Patients were enrolled with a confirmed diagnosis of non-methadone OIC for chronic non-cancer-related pain, who were having fewer than three spontaneous bowel movements (SBMs) per week and experiencing symptoms of constipation. Patients had a mean age of 52, weight of 86 kg and 1.4 SBMs per week. Both PAC-QOL and EQ-5D-3 L data were collected. All patients had at least one medical diagnosis that led to their opioid use. In general, these diagnoses were musculoskeletal in origin, as shown in Table 1.

**Table 1:** Summary of medical diagnoses in study 1033

Diagnosis group	Total (*n* = 439)
Arthralgia	57 (13 %)
Arthritis	47 (10.7 %)
Back pain	228 (51.9 %)
Fibromyalgia	51 (11.6 %)
Intervertebral disc degeneration	110 (25.1 %)
Muscle spasms	53 (12.1 %)
Musculoskeletal pain	27 (6.2 %)
Neck pain	63 (14.4 %)
Osteoarthritis	121 (27.6 %)
Spinal column stenosis	85 (19.4 %)
Other	439 (100 %)

Note: Numbers sum to more than 100 % as patients may have more than one condition

### EQ-5D-3 L – a generic measure of health status

The EQ-5D-is widely used in health care and in clinical research. As a preference-based instrument, the EQ-5D-3 L is a recommended measure for use in health economic evaluations. It takes the form of a descriptive profile evaluation on five dimensions (mobility, self-care, usual activities, pain/discomfort and anxiety/depression). Each dimension is scored by the patient, with 1 indicating no problems, 2 indicating some problems, and 3 indicating extreme problems. The total profile is valued with validated tariffs, resulting in preference-based utility scores that can be used in economic evaluations – the UK tariff was used in this study [12]. The final component of the EQ-5D-3 L is the Visual Analogue Scale (VAS), a fixed height bar on which participants are asked to mark their self-rated health on a scale from 0 (‘worst imaginable health state’) to 100 (‘best imaginable health state’). The VAS, whilst collected in Study 1033, is not widely used in the UK and was therefore not used in our analysis [13].

### The PAC-QOL – a disease-specific instrument

In contrast with generic health instruments such as the EQ-5D-3 L, the PAC-QOL is a disease-specific instrument for patients with constipation developed by Marquis et al. [9]. The PAC-QOL questionnaire provides a standardised and validated assessment of the burden of constipation on patients’ everyday functioning and well-being.

The questionnaire includes 27 questions, which cover 12 symptoms (identified from patient responses). These 12 symptoms are then divided into four subscales (worries and concerns, physical discomfort, psychosocial discomfort, and satisfaction). Participants rate the applicability of each question over the previous 2 weeks by selecting one of 5 boxes (broadly ranging from ‘Not at all’, to ‘All of the time’). The scores for each question are recoded as scores of 0–4, with lower scores indicating fewer problems. Symptom scores and sub-scores are then calculated, as averages of the relevant questions, and symptoms, and the overall score computed as the average score across the 12 symptoms.

### Mapping between PAC-QOL and EQ-5D-3 L – the approach by Parker et al

Parker et al. estimated the relationship between the generic EQ-5D-3 L and the disease-specific PAC-QOL score in a severe CIC population [10]. The EQ-5D-3 L was not directly measured in the study; instead the values were mapped from a different instrument (the SF-36) using the algorithm from Rowen et al. [14]. Three mapping formulas were presented: one formula using only the summary PAC-QOL score as an independent variable, and two formulas using the PAC-QOL score and the PAC-SYM score (a different questionnaire, the Patient Assessment of Constipation Symptoms) as independent variables. The statistic used to test the fit of the mapping formulas was the root mean square error (RMSE). We tested only the mapping between the EQ-5D-3 L and the PAC-QOL, as the PAC-SYM was not collected in the Study 1033.

### Novel mapping formula

In addition to testing the validity of the mapping published by Parker et al., we attempted to re-estimate the parameters observed in the mapping using patient level data from Study 1033. Two mapping formulas were analysed: the relationship between the EQ-5D-3 L and the PAC-QOL total score (as in Parker et al. [10]); and the relationship between the EQ-5D-3 L and PAC-QOL subscale scores.

The statistics used to test the fit of the mapping formulas were the RMSE and the adjusted R^2^ as well as predicted versus observed plots. Mean utility and PAC-QOL scores are presented as mean ± standard deviation. All analyses were performed using the statistical package R.

## Results

### Utility scores from study 1033

**Fig. 1:**
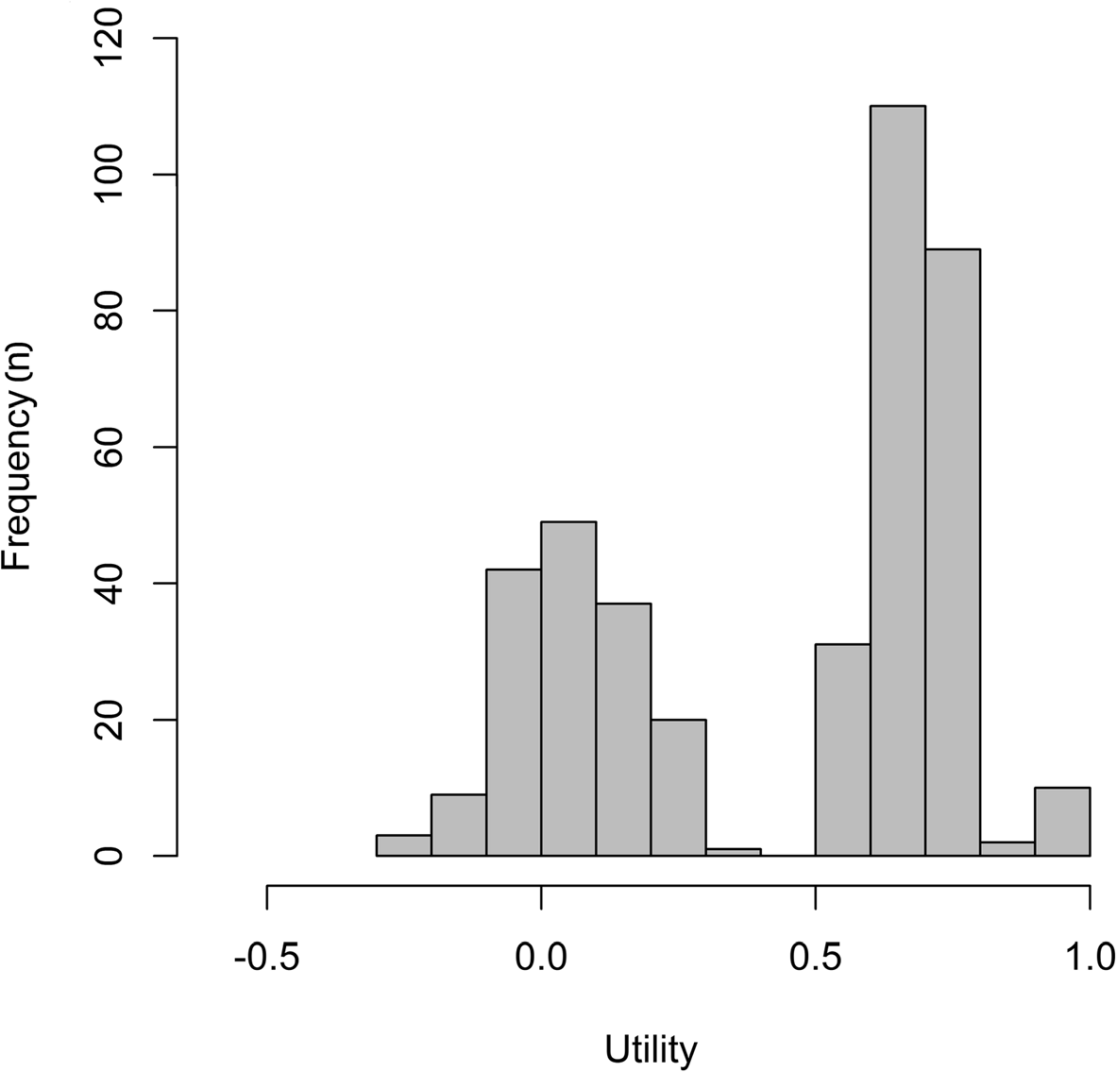
Histogram of measured EQ-5D-3 L utilities in Study 1033

**Fig. 2:**
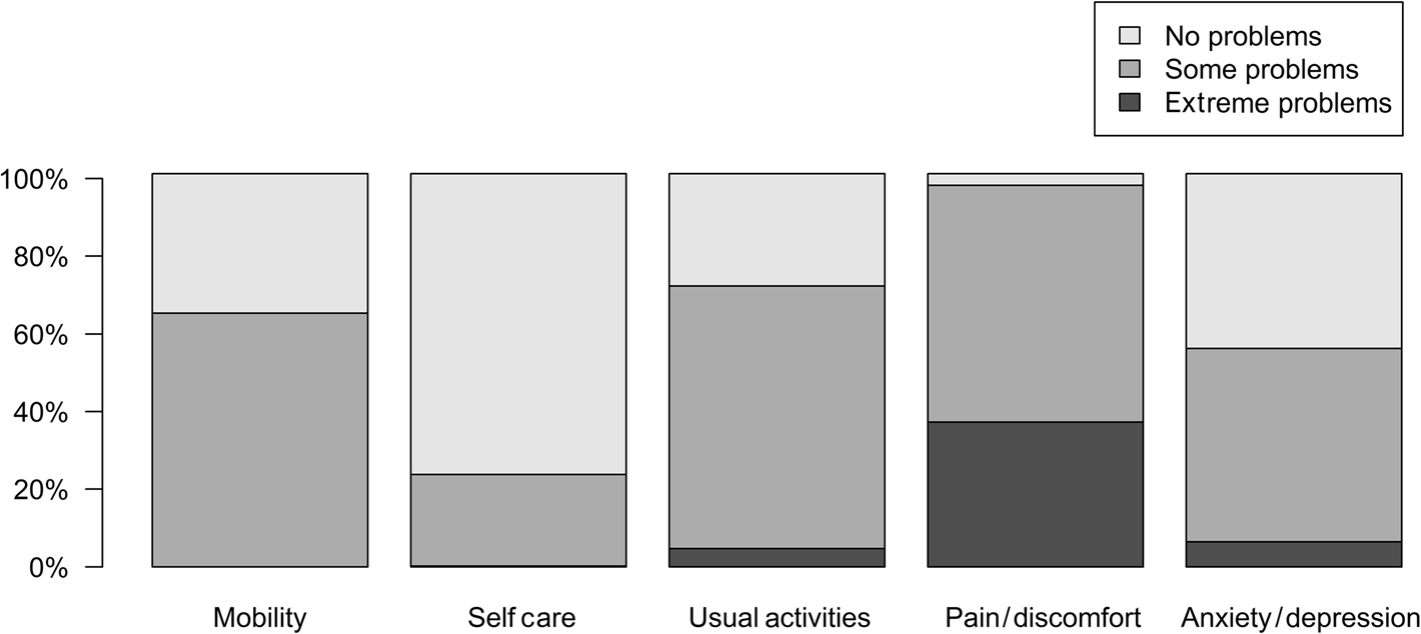
Percentage of patients reporting different levels in the EQ-5D-3 L dimensions

A total of 439 patients with OIC were included in Study 1033, with all except one patient completing the EQ-5D-3 L (*n* = 438, 99.8 %). Figure 1 shows the distribution of EQ-5D-3 L scores, which were distinctly bimodal. The mean utility was 0.450 ± 0.329, with a median of 0.620 and a range from -0.239 to 1. Analysis of the dimension scores showed that severe problems were primarily encountered by patients in the pain/discomfort dimension of the EQ-5D-3 L (Fig. 2).

PAC-QOL scores were measured in all patients in Study 1033, and showed an approximately normal distribution with a mean overall score of 2.462 ± 0.651, a median overall score of 2.495 and a range from 0.739 to 3.938. The most severely impaired PAC-QOL subscore was ‘satisfaction’, while the fewest problems were found on the psychosocial subscore.

The primary efficacy endpoint in Study 1033 was the overall SBM response rate, defined as having three or more SBMs for at least 9 of 12 weeks, and at least one additional SBM over mean baseline SBM during every treatment week. At the end of treatment, patients with three or more SBMs and not using rescue medication in the previous week showed a higher utility than patients with fewer than three SBMs (0.46 ± 0.40 versus 0.34 ± 0.36, *p* = 0.012, Table 2). Similarly, overall PAC-QOL scores were lower, indicating better health, in patients with three or more SBMs compared to those with fewer than three (1.21 ± 0.81 versus 2.09 ± 0.78, *p* < 0.001, Table 2).

**Table 2:** EQ-5D-3 L utility and PAC-QOL by spontaneous bowel movements per week

SBMs at EOT visit^a^	EQ-5D-3 L utility (mean ± sd)	PAC-QOL
≥3	0.463 ± 0.356	1.211 ± 0.812
<3	0.395 ± 0.335	2.091 ± 0.784

Note: ^a^patients using rescue medication in the previous week were classified as having fewer than three SBMs

Key: *EOT* end of treatment, *SBMs* spontaneous bowel movements; sd, standard deviation

### Testing published mapping between EQ-5D-3 L and PAC-QOL, and re-estimating parameters

The mapping formula given by Parker et al. [10] is:

Formula 1:

$$ \mathrm{E}\mathrm{Q}\hbox{-} 5\mathrm{D}\hbox{-} 3\mathrm{L} = 0.977\ \hbox{--}\ 0.098 \times PAC\mathit{\hbox{-}}QOL $$

**Fig. 3:**
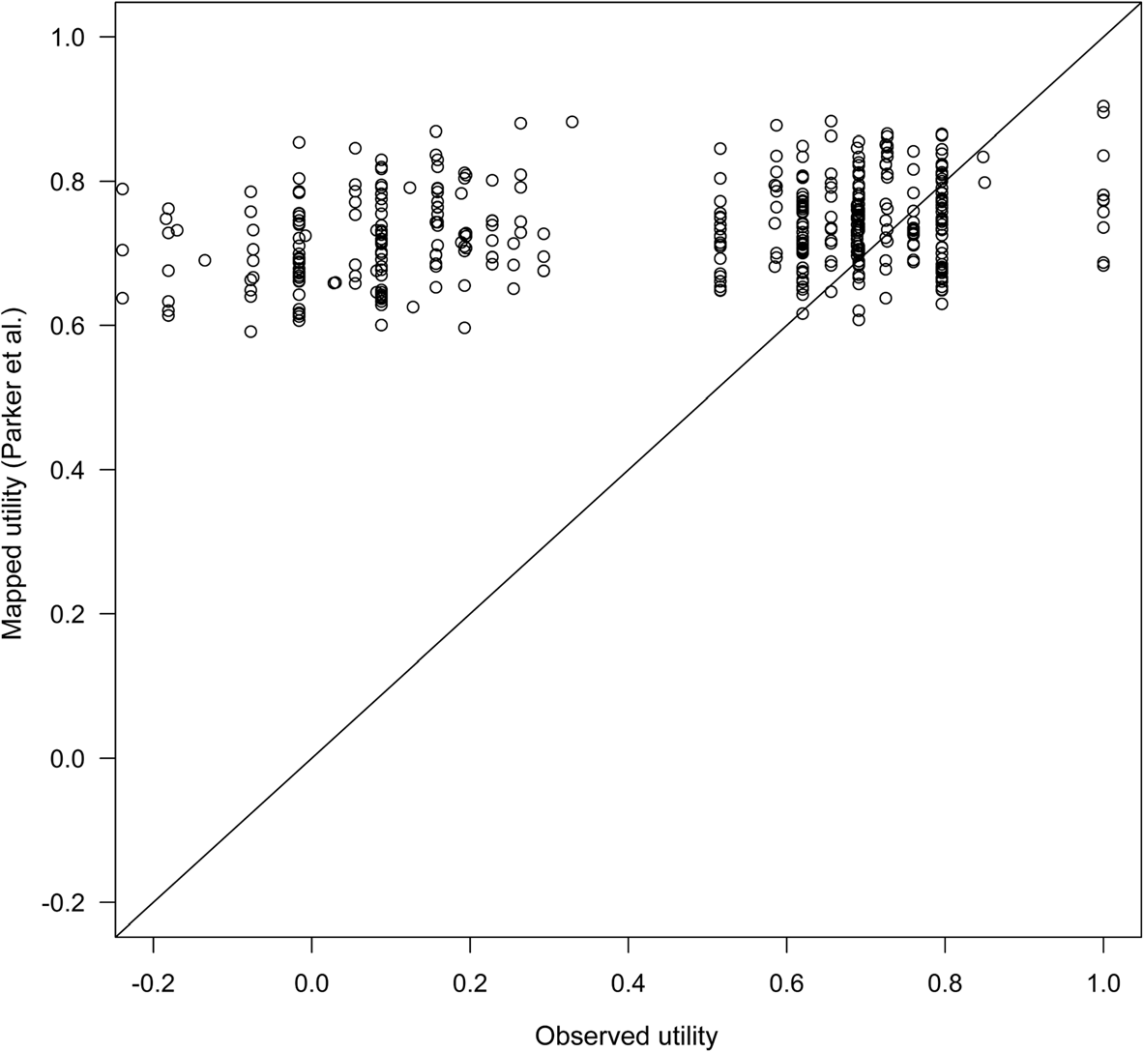
Observed EQ-5D-3 L utility in Study 1033 compared to EQ-5D-3 L utility predicted by mapping from Parker et al. [10]

When applying this formula, the predicted EQ-5D-3 L compared poorly with the measured EQ-5D-3 L in Study 1033, shown in Fig. 3. In particular, low utilities were severely overestimated by the formula, and the mean predicted utility was 0.74, much higher than the measured utility of 0.45. The RMSE was 0.428, while the RMSE reported by Parker et al. was 0.146.

We attempted to re-estimate the equation using the patient level data from Study 1033, using a generalised linear model in R to map the EQ-5D-3 L utility to the PAC-QOL overall score. A second re-estimation was then attempted using the same regression method, but using the EQ-5D-3 L and the PAC-QOL subscales, which then had interaction terms between all subscales added to them. The results of these analyses showed that there was a negative but highly variable correlation between the PAC-QOL and the EQ-5D-3 L, with all models showing a poor fit to the data. Estimating EQ-5D-3 L using the PAC-QOL score as the only independent variable resulted in the following formula:

Formula 2:

$$ \mathrm{E}\mathrm{Q}\hbox{-} 5\mathrm{D}\hbox{-} 3\mathrm{L} = 0.780\ \hbox{-}\ 0.134 \times PAC\mathit{\hbox{-}}QOL $$

**Fig. 4:**
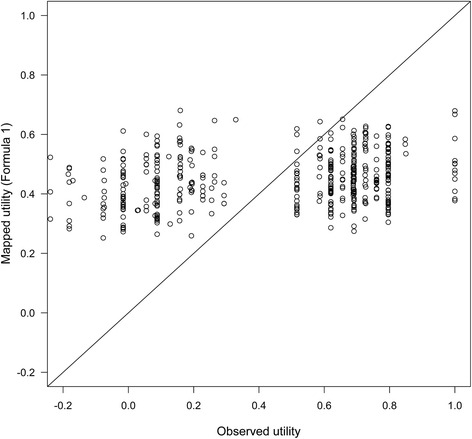
Predicted and observed EQ-5D-3 L utility from Study 1033

The RMSE was 0.317 and the adjusted R^2^ was 0.068, indicating a weak association between the PAC-QOL and the EQ-5D-3 L. The mapping showed a poor fit to the data; the high utilities were underestimated, and the low utilities were overestimated (Fig. 4). Attempting a mapping using the PAC-QOL subscales as independent variables yielded:

Formula 3:

$$ \mathrm{E}\mathrm{Q}\hbox{-} 5\mathrm{D}\hbox{-} 3\mathrm{L} = 0.716 + 0.023 \times Satisfaction\hbox{--}\ 0.091 \times Physical + 0.013 \times Psychosocial\hbox{--}\ 0.062 \times Worries $$

The RMSE was 0.314 and the adjusted R^2^ was 0.080, which, although an improved fit compared to the mapping using the PAC-QOL total score, still indicates a weak association between the PAC-QOL and the EQ-5D-3 L. The correlation plot was similar in appearance to Fig. 4, where the high utilities were underestimated and the low utilities were overestimated. Furthermore, mapping did not significantly improve when interaction terms were added between the subscale scores or when alternative mappings were estimated in ranges of the PAC-QOL score.

## Discussion

The first notable finding of the analysis of EQ-5D-3 L at baseline was the level of patient utility. This low utility is consistent with the existing literature, where patients report similar utilities [4–6].

The mean utility of 0.450 is exceptionally low; it is lower than in patients with comparable symptoms in CIC [10] and even lower than the majority of EQ-5D-3 L estimates in patients with advanced cancer, a condition that we would have expected to be much more severe [15]. The main source of the low utility was the pain dimension, shown by the high scores on the pain/discomfort dimension of the EQ-5D-3 L scored by patients in Study 1033 (Fig. 2). This could be explained by the comorbid conditions that resulted in long-term opioid therapy – in Study 1033, the majority of patients were suffering from several different diagnoses which all cause rheumatic pain or pain of the musculoskeletal system (Table 1).

Although there was an association between high PAC-QOL scores and lower EQ-5D-3 L utilities, it was not possible to reliably map the PAC-QOL on to the EQ-5D-3 L; the mapping formula provided by Parker et al. proved unreliable in our study population [10]. A possible explanation for the failure of the mapping exercise is that although the outcomes are the same (utility and PAC-QOL scores), the trials were conducted in different populations; the study by Parker et al. was in CIC, while Study 1033 was in OIC with chronic non-cancer pain. Although patients in both studies had a similar level of constipation severity (as measured by the number of spontaneous bowel movements), all patients in the group receiving opioids had comorbid conditions leading to the opioid use. This may have led to the poor scores on the EQ-5D-3 L pain dimension. A second possible explanation may be that Parker et al. did not directly measure the EQ-5D-3 L in their study, but instead measured the SF-36, which was in turn mapped to the EQ-5D-3 L. This two-step approach may have introduced a different relationship between the PAC-QOL and the EQ-5D-3 L, although the differences between patient populations would have remained.

The mapping formulas directly estimated in this study performed slightly better than the formula provided by Parker et al. However, these formulas still performed poorly compared to other published mapping studies [8, 16], as shown by the high RMSE and low R^2^ scores. Therefore, we would not recommend their use; the mapping algorithms we used consistently underestimated high utilities and overestimated low utilities, despite the multiple methods attempted to obtain a better fit. As such, the most likely explanation is that, in this population, other factors, which are not captured by the PAC-QOL, are the determinants of quality of life (as measured by the EQ-5D-3 L utility). Similar conclusions have been drawn for mapping studies with comparable instruments such as the Over-Active Bladder Questionnaire [8]. Finally, while the mapping formula of Parker et al. appeared to perform better in their population of CIC (as demonstrated by a lower RMSE statistic), other important fit criteria such as the R^2^ and predicted versus observed plots were not presented. Therefore, under/over-prediction cannot be assessed.

## Conclusion

Mapping is a commonly used technique in the field of health economics to derive generic utilities when only disease-specific measures are available. In this analysis, we applied an existing mapping between two instruments to a related disease area. As a result, we showed that the original mapping was a poor fit, and re-estimation proved unsuccessful. In the absence of directly measured patient utilities, caution should be exercised with regard to the generalisability of mapping instruments in this area. While the mapping by Parker et al. in CIC appears to demonstrate a good fit between the PAC-QOL and the EQ-5D-3 L in CIC [10], we found no such relationship in OIC.

However, our analysis shows that OIC patients with chronic non-cancer pain exhibit a very low level of utility, as consistently seen across the literature. It is likely that the observed values relate not only to the condition under investigation (OIC) but also to the underlying health issues for which opioids are used. Regardless of origin, the low quality of life of patients should be acknowledged.

Further research on the validity of mapping algorithms is recommended, both in different datasets within the same disease area (as validation), but also in related disease areas where the same instruments are used (as has been done with the EORTC-QLQ-C30 and EQ-5D-3 L [17]). Such work would ensure that published algorithms are reproducible and give reliable results for use in health economic evaluations.
